# Investigation of the relationship between incidence of mental disorders and economic growth among the Visegrad countries

**DOI:** 10.3389/fpubh.2022.982716

**Published:** 2022-09-07

**Authors:** Gergő József Szőllősi, Klára Boruzs, Andrea Karcagi-Kováts, Nándor Kalas, Gábor Bányai, Klára Bíró

**Affiliations:** ^1^Faculty of Health Sciences, University of Debrecen, Debrecen, Hungary; ^2^Faculty of Economics and Business, Institute of Health Economics and Management, University of Debrecen, Debrecen, Hungary; ^3^Faculty of Economics and Business, Department of Environmental Economics, University of Debrecen, Debrecen, Hungary; ^4^Doctoral School of Health Sciences, University of Debrecen, Debrecen, Hungary

**Keywords:** mental health, mental disorder (disease), health economic, Visegrad countries (V4), economic growth

## Abstract

Prevention and care for mental disorders represent an important public health task in achieving global development goals. Proper access to adequate healthcare and social services is an important step related to care for mental disorders, which is presumably strongly related to economic growth. The main aim of the study was to investigate the relationship between the economic growth and the incidence of mental disorders in the V4 countries. An ecological correlation study was conducted regarding the four Visegrad countries. Indicators were derived from the World Health Organization (WHO) ‘Health for All' (HFA) online database and Penn World Table version 10. The incidence of mental disorders increased in the V4 countries throughout the years between 2000–2018 except in Hungary, where a decreasing trend was observed. The prevalence of mental disorders increased in all countries as well, but it stagnated in Hungary. At the same time standardized death rate due to mental disorders increased in all Visegrad countries. According to the Hungarian data, while the prevalence of the disease did not change remarkably, the incidence decreased and the mortality increased as well as the prescription of drugs used in the treatment of mental disorders. This could indicate a serious hidden morbidity.

## Introduction

Prevention and care for mental disorders represent an important public health task in achieving global development goals (1–3). Mental illnesses - often known as mental health disorders - covers a broad category of illnesses that could impact emotion, thought, and behavior (4). This could include depression, anxiety disorders, schizophrenia, eating disorders and addictive behaviors (5). Many people have mental health concerns from time to time during their lifetime, but a mental health concern could easily become a more severe mental illness when its signs and symptoms cause frequent health-related problems which could affect the individuals' functionality (3, 4, 6). Lot of factors could contribute to the development of mental health disorders, for instance anxiety, – self-esteem issues, anger or grief, therefore there could be many distinguished mental health problems, with many different manifestations (4). The possible risk factors which could lead to mental disorders are sometimes considered as comorbidities or consequences of the disorders (7). Effective strategies are available for preventing mental disorders, such as consulting a mental health specialist, seeking help from a primary care provider or contacting somebody in the individual's faith community (8, 9). Effective treatments are available in order to alleviate the burden related to mental health problems (4, 10). Both children (e.g., through protection and psychological support) and adults have demonstrated to benefit from prevention programs (e.g., through psychosocial assistance after disasters and conflicts) (11, 12). There are also therapies that are effective, such as cognitive behavior therapy or psychotherapy, which can effectively cure mild to moderate depression (Health Quality Ontario), (13). Antidepressants can be an effective treatment for mild-to-moderate depression, however, they should not be used as the first line of therapy for mild depression (14). They should not be used to treat depression in children, and they should not be used as the first line of treatment in teenagers, who should be treated with special caution. Even though many mental health illnesses could be adequately treated at a reasonable cost, the gap between those who need care and those who receive care remains significant (15, 16). Increased investment is needed on all fronts: to raise mental health awareness and eliminate stigma; to promote access to quality mental health care and effective therapies; and to conduct research to discover new treatments and improve existing treatments for all mental diseases (17–19). Furthermore, proper access to adequate healthcare and social services is an important step in the care of mental disorders, which is presumably strongly related to economic growth (20–22). However, there are no data related to the relationship between economic growth and the incidence of mental health problems in the Visegrad countries (V4), even though the 10^th^ International Conference of the Visegrad Platform of Eurodiaconia focused on reinventing the approach toward psychiatry. Attendees debated how different organizations and institutions can aid the integration of people with mental illnesses into society (23). The V4 countries share a similar historical, socio-economic background, that is why public health interventions and care focusing on mental health problems could be approached similarly (24). That is why the relationship between the economic growth and the incidence of mental disorders should be the same. However, studies with V4 countries data were published regarding the determinants of financial indebtedness of enterprises, earnings management practices, analysis of the development of earnings of transport sector and tax aspects of mining companies, but there is a lack of studies which focus was to assess the relationship between mental disorders and economic growth (25–29). Furthermore, economic growth could be associated with several factors, for example with foreign direct investments or entrepreneurship activities, but there is no data regarding the relationship with mental diseases (30, 31). Thus, the purpose of this study was to investigate the relationship between economic growth and the incidence of mental disorders.

## Aim

The main aim of this study was to investigate the relationship between the economic growth [expressed as Gross Domestic Product (GDP) per capita] and the incidence of mental disorders by fitting second-degree polynomial curves in the V4 countries followed with a special focus on the Hungarian situation.

## Methods

An ecological correlation study was conducted regarding the four Visegrad countries. Indicators derived from the World Health Organization (WHO) “Health for All” (HFA) online database. This data could be used for monitoring basic demographics, health status, health care resources and expenditures in the European Region. Data from HFA were downloaded in MS Office Excel format (.xls). The incidence of mental disorders per 100 000 persons was used in the analysis, the indicator code was E991201.T. This indicator represents the standardized rate of patients with newly diagnosed mental disorders for the first time in their life. The data for Hungary were from the “Register of Psychiatric and Child Psychiatric Care Facilities of the National Institute of Psychiatry (OPNI) and the Register of the Addictology Centers of the National Alcohological Institute (OAI)”. Data from Slovakia were from the “National Health Information Center (NIC)”; data from the Czech Republic were from the “Institute of Health Information and Statistics of CR (IHIS CR)”. While data related to the incidence of mental health disorders in Poland were from the Institute of Psychiatry and Neurology. Prevalence of mental disorders was expressed as percentage (%). Standardized death rate (SDR) for mental disorders were expressed per 100 000 persons. Data regarding GDP were taken from the “Penn World Table” (PWT) version 10., which is an online database containing information regarding income, output and input for 183 countries between 1950 and 2019. The database covered the expenditure-side real GDP at chained PPPs (32), to compare relative living standards across countries and over time and countries' populations (in millions). These two were divided in order to express the GDP per capita, followed by the investigation of the prescription of subsidized Vortioxetin, Trazodon and Fluoxetin in Hungary over a 13-year period between 2008 and 2021. Data were obtained from the drug utilization database available on the website of the National Health Insurance Fund Management (NEAK). These reports are based on NEAK's prescription data and are added to an online database on the website after the data have been processed. Drug use was interpreted using the DOT value. DOT is an abbreviation for days of therapy or days of treatment, a naturalistic indicator of the evolution of drug use, which is used to equalize different pack sizes of drugs. The DOT value shows how many days of therapy each drug is sufficient for. It is calculated for a given product by dividing the total active ingredient in the package, measured in milligrams, by the defined daily dose (DDD), also measured in milligrams (33). The DDD is an indicator of the average daily dose of an active pharmaceutical ingredient calculated by the World Health Organization (WHO) based on reports from pharmaceutical manufacturers. All data were plotted using line charts. In order to analyse the relationship between economic growth and the incidence of mental disorders, second-degree polynomial curves were fitted related on the incidence of mental disorders related to economic growth. Determinant coefficients were also calculated to measure the goodness-of-fit. Full dataset for GDP and the incidence of mental disorders indicator for the V4 countries were available for the period of 2000–2018, that is why this timeframe was chosen in this analysis.

## Results

### Poland

**Figure 1:**
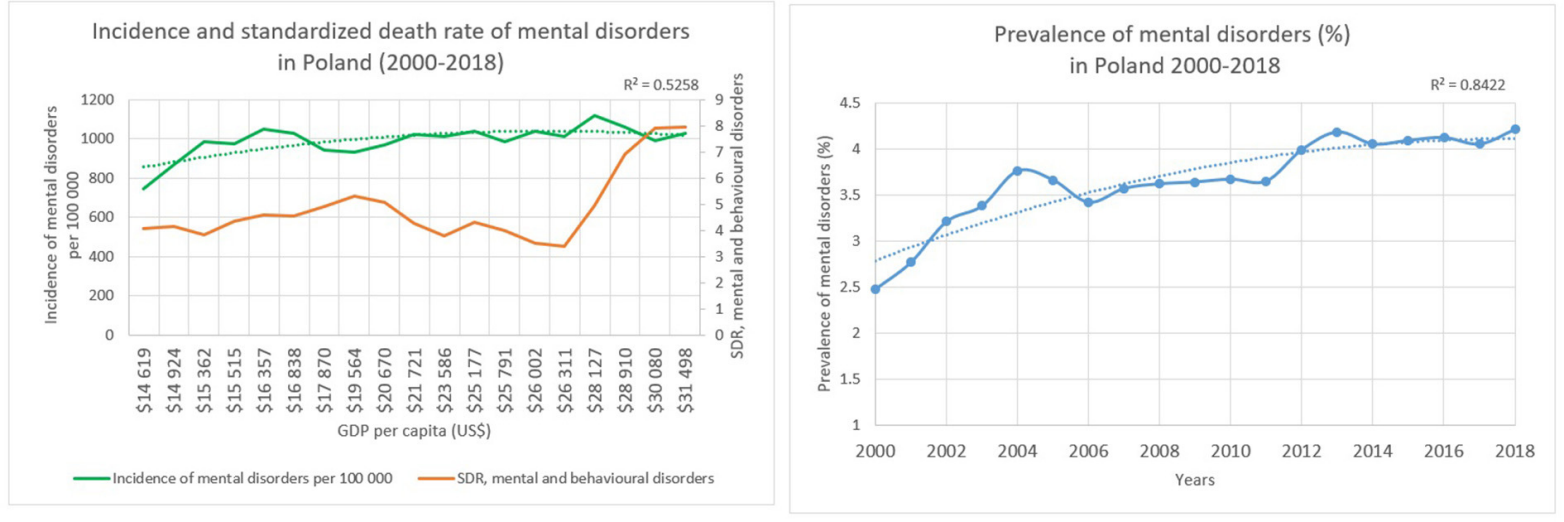
Characteristics of Poland.

GDP as the sum of the value of all goods and services produced by Poland varied between US$ 14,619 and US$ 31,498 per capita between 2000 and 2018. The incidence of mental disorders showed a slightly increasing trend in the country, whereas the incidence was equal to 746 per 100 000 persons in 2000 and it increased to 1,028 per 100 000 persons by 2018. The prevalence of mental disorders was approximately 2% in 2000 which increased to 4% during the years with a determination coefficient of 0.7304. At the same time standardized death rate related to mental and behavioral disorders showed an increasing trend. Standardized death rate approximately doubled throughout the years, as it was equal to 4.1 per 100 000 persons in 2000 and it increased to 7.9 per 100 000 persons by 2018. Point of intersection could be observed regarding the incidence and mortality, when the GDP was between US$ 28,910 per capita and US$ 30,080 per capita with an incidence of 1,060 and 988 per 100,000 persons in 2016 and 2017. Regarding the second-degree polynomial curve fitted on the incidence of mental health disorders and GDP, the determinant coefficient describing the relationship was 0.5258 in Poland, hence the relationship could be described as 52% of movements of incidence related to mental disorders is explained by economic growth (Figure 1).

### Czech Republic

**Figure 2:**
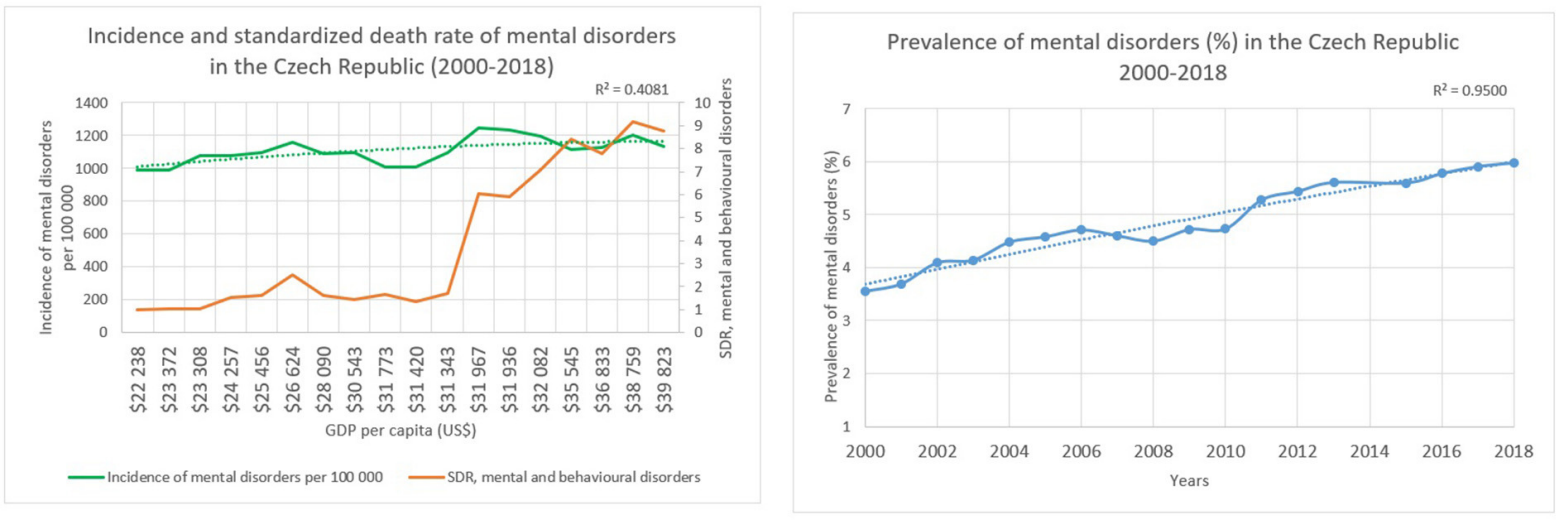
Characteristics of the Czech Republic.

The GDP in the Czech Republic diverged from US$ 22,238 and US$ 39,823 per capita. The same increasing trend could be observed in the country regarding the incidence of mental disorders as seen in Poland, therefore it increased from 992 to 1,136 per 100 000 persons between 2000 and 2018. While the standardized death rate related to mental and behavioral disorders showed an increasing trend as well, with a rate of 1.0 in 2000 and 8.8 per 100,000 persons. The prevalence of mental disorders changed from 4 to 6% between 2000 and 2018 with a determination coefficient of 0.9500 regarding the years. Intersection points could be identified related to the indicators covering incidence and mortality between 2015 and 2016, where the GDP was US$ 35,545 and US$ 36,833 per capita, respectively. The determinant coefficient describing the relationship between the incidence of mental diseases and economic growth with a help of a second-degree curve was equal to 0.4081 in the Czech Republic. This means that 41% of the variability could be explained regarding the incidence of mental disorders and economic growth (Figure 2).

### Slovakia

**Figure 3:**
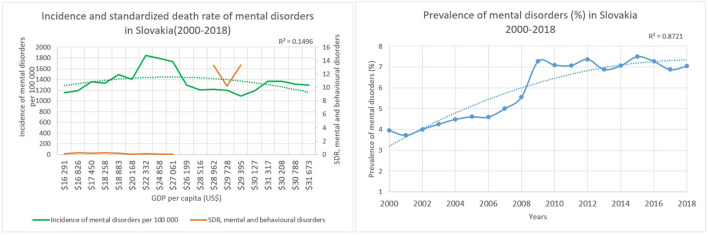
Characteristics of Slovakia.

In Slovakia the GDP ranged between US$ 16,291 and US$ 31,673 per capita during 2000 and 2018. The incidence of mental disorders slightly increased from 1,152 in 2000 to 1,292 per 100,000 persons in 2018. Mental health disorders' prevalence was 4% in 2000 and 7% in 2018 with a determinant coefficients value of 0.8721 regarding the year variable. At the same time the standardized death rate related to mental and behavioral disorders showed an increasing trend, however there were no full data regarding this indicator. Although, a huge increase could be seen related to mortality, because the standardized death rate was 4 per 1 000 000 persons in the 2000s, which decreased to 2 per 1 000 000 persons in 2005, and later climbed to 134 per 1 000 000 in 2014. The determinant coefficient describing the relationship regarding the incidence of mental diseases and economic growth was 0.1496 in Slovakia (Figure 3).

### Hungary

**Figure 4:**
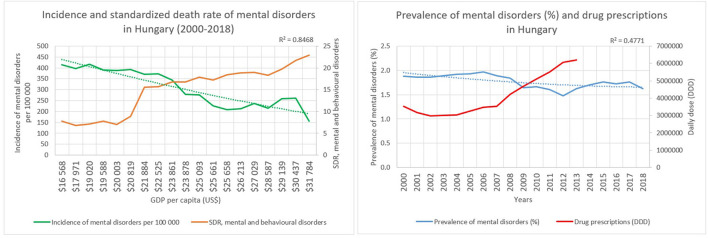
Characteristics of Hungary.

GDP varied in Hungary between US$ 16,568 and US$ 31,784 per capita between 2000 and 2018. The incidence of mental disorders in Hungary was 414 per 100,000 persons in 2000; furthermore, a declining trend was observed in the following years, because it decreased to 156 per 100,000 persons in 2018. The prevalence was 2% in Hungary, small changes were observed regarding the prevalence (1.5–2.0%), but overall, it could be considered as stagnating with a 0.4771 determination coefficient toward prevalence of the disorders in the studied years. The standardized death rate related to mental and behavioral disorders showed an increasing trend over the years, which was 7.7 in 2000 and increased to 22.9 in 2018 per 100 000 persons. A point of intersection regarding incidence and mortality of mental disorders could be seen in 2008, where the incidence was equal to 344 and the standardized mortality was 16.76 per 100,000 persons, with the GDP being US$ 23,861 per capita. The determinant coefficient was equal to 0.8468 related to the second-degree polynomial curve, which means a highly reliable model for future forecasting of the relationship regarding economic growth and incidence of mental disorders in Hungary. More precisely, according to the model it seems that 85% of the incidence of mental disorders could be predicted by economic growth. Further analysis was done regarding the Hungarian drug prescription for mental disorders between 2008 and 2021, according to which an increasing trend could be seen in Hungary (Figure 4).

## Discussion

In its opinion in the Green Paper, the European Economic and Social Committee made it clear that mental health in Europe is a major issue. It was found that mental disorders are among the top ten causes of disability worldwide and, as a result, has devastating social and economic consequences for individuals, families and governments. Furthermore, there is an increasing need for a community-level mental health strategy reformulation and implementation. In recent years several trends were observed: an explosive growth in the demand as it is becoming a mass phenomenon; a paradigm shift regarding mental health; and the irreversible appearance of representatives of stakeholders. All these aspects entailed changes in legislation and regulation at a different rate in countries, indicating that the political and administrative system had become aware of the problem (34). No data are available on the relationship between economic growth and the incidence of mental health problems in the Visegrad countries. As the V4 countries share a similar historical, socio-economic background, the changes taking place in these countries may be important in assessing the effectiveness of prevention and care services in the field (24). The results showed that Slovakia, the Czech Republic and Poland have a weaker relationship in terms of incidence, prevalence of mental disorders and economic growth compared to Hungary. Hungarian results showed that as the economic growth increased, the incidence of mental illnesses decreased, in contrast, for GDPs above US$ 23,000, the gap starts growing in terms of incidence and mortality regarding mental illnesses, while the prevalence of mental illness remains almost the same. Further aggravates the fact that the drug prescription increased during the study time frame as well. According to this research, it seems to be an essential step to examine the institutional changes that have taken place in Hungary. The National Institute of Psychiatry and Neurology dealing with the vast majority of mental illnesses was closed down in 2007, which may have contributed to the hidden burden and morbidity of mental disorders, therefore patients in need of care could go undetected, which could further complicate the burden of mental disorders. For the first time, a State Audit Office examined the situation of psychiatric inpatient care between 1 January 2006 and 30 September 2011, including changes that had occurred at the end of the audit. The purpose of the audit was to assess whether the resources spent on the transformation of psychiatric care were used properly and whether the transformation resulted in more cost-effective and higher quality, more accessible care. The audit looked at the situation of psychiatric inpatient care and found a dismal situation. Deteriorating standards of care, declining capacity and declining care indicators were all reflected in the findings. Overall, the situation assessment found that unfavorable conditions of psychiatric health care have changed both for the institutional system and for patients as well. The capacities of health and social psychiatric services are not coordinated and are not based on morbidity data in the absence of disease registers (35). Capacities of health and social psychiatric care are not well coordinated, and in the absence of disease registries, are not based on morbidity data. The report states that although the number of beds reserved for chronic patients has increased slightly but the number of active beds decreased, and there are fewer and fewer doctors and health care professionals working in this field. The audit recommended that, as soon as possible, a system of care should be put in place to ensure equal access for all concerned (35). In 2018 five national health programmes were launched, to which two more programmes are connected, the primary care policy programme and the public health policy programme, and a large national institute has been set up behind each major programme. As a result, the National Institute of Mental, Neurological and Neurosurgery was established, which is primarily responsible for the mental health program. The new institution was created by merging the National Institute of Clinical Neuroscience and the National Institute of Psychiatry and Addiction (35). The difficult and complex issue of mental illnesses is likely to have been exacerbated by the emergence of the COVID-19 pandemic and the associated patient care restrictions, not to mention the now known complications and post-covid conditions of the disease (36, 37). The question for the future will be how all this affected the situation of psychiatric care in Hungary, and thus the individual and social burdens caused by mental illness.

## Conclusion

According to the results observed in this study, it seems that the incidence of mental disorders increased in the V4 countries related to economic growth between 2000–2018 with the exception of Hungary, where a decreasing trend regarding the incidence was observed. Despite of this the standardized death rate due to mental disorders increased in all Visegrad countries. The prevalence of mental disorders increased in all countries participating in the analysis, but it stagnated in Hungary, which could contribute to discrepancy. According to the Hungarian data, while the prevalence of the disease did not change significantly, the incidence decreased, while the mortality increased as well as the prescription of drugs used in the treatment of mental disorders. This could indicate a serious hidden morbidity related to mental disorders in Hungary. In order to alleviate the burden of the undetected mental health problems, the healthcare providers in Hungary should focus even more on patients with possible risk factors regarding mental health problems.

## Strengths and limitations

This study could be considered as unique due to its topic and the methods applied, and the possible relationship between the variables of interests could be observed; however, the drawn conclusions should not be interpreted on individual level data in order to prevent ecological fallacy. In addition there is no data regarding drug prescriptions from other countries, which could be considered as a weakness of this study. The Health for All explorer uses interactive online tools in order to visualize regional and national differences related to health indicators, therefore all data, graphs, charts and maps could be exported and are available with no charges for publication.

## Author's note

No assistance in the preparation of this article is to be declared. No previous presentations were held from this topic.

## Data availability statement

The original contributions presented in the study are included in the article/supplementary material, further inquiries can be directed to the corresponding author/s.

## Author contributions

GS: conceptualization, methodology, data analysis, and writing-original draft. KBí: conceptualization, methodology, formal analysis, data curation, writing-original draft, and supervision. GB and KBo: writing-review and editing. AK-K and NK: conceptualization, methodology, and writing-review and editing. All authors contributed to the article and approved the submitted version.

## Conflict of interest

The authors declare that the research was conducted in the absence of any commercial or financial relationships that could be construed as a potential conflict of interest.

## Publisher's note

All claims expressed in this article are solely those of the authors and do not necessarily represent those of their affiliated organizations, or those of the publisher, the editors and the reviewers. Any product that may be evaluated in this article, or claim that may be made by its manufacturer, is not guaranteed or endorsed by the publisher.
